# Defining malnutrition in persons with spinal cord injury – does the Global Criteria for Malnutrition work?

**DOI:** 10.29219/fnr.v68.9989

**Published:** 2024-03-25

**Authors:** Hanne Bjørg Slettahjell, Maria Bastakis, Fin Biering-Sørensen, Vegard Strøm, Christine Henriksen

**Affiliations:** 1 Sunnaas Rehabilitation Hospital, Bjørnemyr, Norway;; 2 Department of Nutrition, Institute of Basic Medical Sciences, University of Oslo, Norway;; 3 Department of Clinical Medicine, University of Copenhagen, Copenhagen, Denmark;; 4 Department for Brain- and Spinal Cord Injuries, Bodil Eskesen Center, Glostrup, Denmark

**Keywords:** spinal cord, rehabilitation, malnutrition, fat-free mass

## Abstract

**Background and aims:**

Physiologic and metabolic changes following spinal cord injury (SCI) lead to an increased risk of malnutrition. The Global Leadership Initiative on Malnutrition (GLIM) is a three-step approach to diagnose malnutrition: 1) screening; 2) phenotypic and etiological criteria; and 3) malnutrition severity. The main aim of this study was to assess malnutrition in patients with SCI, according to the GLIM criteria.

**Methods:**

Patients with SCI (≥ 18 years) admitted to rehabilitation were included. Anthropometrics, food intake, and inflammation were assessed on admission. Fat-free mass index (FFMI) was estimated from bioimpedance analysis. Malnutrition was diagnosed by the GLIM criteria, using the Malnutrition Universal Screening Tool (MUST) as the first step screening tool. Sensitivity and specificity analyses were performed.

**Results:**

In total, 66 patients were assessed (50 men) with a mean age of 51.4 (± 17.4) years and median time since injury was 37.5 (10–450) days. The mean body mass index was 24.7 (± 4.2) kg/m^2^, and 1-month involuntary weight loss was 5.7 (± 4.4)%. FFMI for men was 17.3 (± 1.9) and for women 15.3 (± 1.6) kg/m^2^. Forty-one patients (62%) were malnourished according to the GLIM criteria: 27 moderately and 14 severely malnourished. MUST was not able to detect malnutrition risk of nine patients, giving a moderate agreement (kappa 0.66), with a sensitivity of 0.78 and a specificity of 0.92 compared to the GLIM diagnosis.

**Conclusions:**

In this cross-sectional study, 62% of subacute SCI patients were malnourished according to the GLIM criteria. The screening tool MUST showed moderate agreement with the GLIM criteria and did not detect risk of all patients with a malnutrition diagnosis. The clinical implications of these findings need further investigation.

## Popular scientific summary

- According to global criteria for diagnosing malnutrition (Global Leadership Initiative on Malnutrition (GLIM)), the prevalence of malnutrition is high among patients with a subacute spinal cord injury (SCI).
- Loss of fat-free mass is a natural and inevitable consequence of an SCI and contributes to a malnutrition diagnosis.
- One may argue that the SCI itself inflicts a malnourished state of the human body in the acute/subacute phase.
- The validity of the GLIM criteria among patients with a SCI is questioned, and clinical implications need to be investigated.

Metabolic changes after spinal cord injury (SCI) demonstrate an increased level of inflammatory markers combined with a hypometabolic response (1, 2). Muscle catabolism and negative nitrogen balance post-SCI are largely attributed to the consequences of acute motoric and sensory loss, and weight loss is normal during the initial weeks of SCI (2–4). Diagnosing malnutrition following SCI requires special consideration due to muscle atrophy and accompanied weight loss. Disease-related malnutrition is characterized by reduced food intake and involuntary weight loss and is linked to a poorer clinical outcome in terms of recovery, morbidity, and mortality in several patient groups (5, 6). A study on a mixed group of hospitalized patients in Norway (mostly patients from internal medicine and surgery followed by oncology, orthopedics, geriatric, and other) emphasizes that many have a reduced food intake, particularly after surgery (7). Malnutrition may negatively affect rehabilitation after SCI by increasing the risk of pressure injuries, delayed wound healing, infections, respiratory complications, and increased length of stay (8, 9). Identifying patients at risk of malnutrition is important to take appropriate actions to improve nutritional status. Malnutrition assessment and diagnosis associated with disease or injury have lacked global consensus in the clinical setting. Previous studies have used different tools to assess malnutrition risk in the SCI population, including the generic Malnutrition Universal Screening Tool (MUST), SCI-specific screening tools, and dietetic assessment (8–12). These studies report a high risk of malnutrition among individuals with SCI (8, 9, 11–13).

The Global Leadership Initiative on Malnutrition (GLIM) criteria was convened in 2016 and is now the globally recommended scheme for diagnosing malnutrition (14). The GLIM guidelines recommend a three-step model to detect and diagnose malnutrition by assessing phenotypic and etiological criteria. Step 1 includes screening with a validated malnutrition screening tool. MUST is an example of a quick and easy tool often used in clinical practice (15). Step 2 involves evaluating phenotypic criteria; degree of involuntary weight loss, body mass index (BMI), fat-free mass (FFM), and etiological criteria; food intake and inflammation (14). In step 3, a malnutrition diagnosis is set based on the criteria from steps one and two, categorized as no, moderate, or severe malnutrition. FFM is a relatively new criterion in the malnutrition diagnosis. Assessing FFM is important to differentiate lean body mass from adipose tissue, as the negative consequences of disease-related malnutrition are linked to the loss of lean body mass (16). However, few or no studies have assessed the FFM of individuals with SCI and compared this with criteria for malnutrition diagnosis (GLIM).

Several methods to assess FFM exist. In the clinical setting, bioimpedance analysis (BIA) is an available and non-invasive method to assess body composition. BIA differentiates body composition by measuring the electrical water conductivity of tissues, and the accuracy of BIA depends on hydration status and mathematical equations to interpret fat and FFM (17). Increased extracellular water after SCI may influence the prediction of total body water (TBW) from BIA and estimates of FFM (18). However, by using population-specific equations and multifrequency BIA, one can achieve reliable measurements of TBW and FFM. In a study among individuals with subacute SCI, deuterium dilution (DD) and the doubly labeled water method were utilized to calculate TBW and FFM, which were then compared to measurements obtained from dual-energy x-ray absorptiometry (DXA) and several BIA equations (19). They found that a SCI-specific BIA equation developed by Kocina et al. (20) had a good fit, showing a high concordance with DD, a mean bias of 0.6 kg and moderate limits of agreement (LOA) ± 5.2 kg.

The primary aim of the present study was to assess the prevalence of malnutrition according to the GLIM criteria. Explorative aims were to evaluate the agreement between the MUST risk of malnutrition and the GLIM diagnosis. Moreover, we investigate the agreement between the manufacturers BIA equation and the equation developed by Kocina et al. (20).

## Methods

### Participants

Patients ≥ 18 years of age admitted for SCI rehabilitation were included in this cross-sectional study, from October 2018 to March 2023. Patients with congenital disorders, psychological factors, head trauma, poor cognition, lingual barriers, cauda equina, and severe comorbidity (alcohol/substance abuse, cancer, progressive disorders, diabetes, and heart failure) were excluded. Data on the first 18 patients were obtained from of a pilot study, which included a mix of subacute and follow-up non-traumatic and traumatic SCI patients. These data were collected by a master student in clinical nutrition under the supervision of a dietitian (first author: HBS). The remaining samples include baseline data from an ongoing randomized controlled trial among individuals with traumatic SCI admitted for subacute SCI rehabilitation. All the data were collected and quality checked by the dietitian (HBS). Data on hospitalization and subtypes of SCI were obtained from the electronic medical records according to the International Standards for Neurological Classification of SCI, including the American Spinal Injury Associations’ (AIS) impairment scale grade (21).

### Nutritional screening

The Norwegian version of the MUST (22), originally developed by Stratton and colleagues (15), was used for nutritional risk screening (step 1). The nurses at the ward performed nutritional screening with MUST routinely on admission, including assessment of patient’s body weight, BMI, and weight loss (kg) post-injury. This was later quality checked by the dietitian. In cases where MUST was missing, this was performed by the dietitian based on data from the patients’ journal and dietitian interview. Calibrated weight scales at the ward were used, including a wheelchair weight scale, standing weight scale, and a chair weight scale. All weight scales were routinely calibrated to ensure its accuracy. As a standard procedure, the weight of the wheelchair, shoes, and clothes was subtracted to estimate actual body weight. Body height was self-reported by the participant. MUST is a five-step screening tool using a score system to identify adults who are malnourished or at risk of malnutrition. Step 1: calculating and scoring BMI by measuring height and weight. BMI ≥ 20 kg/m^2^ equals 0 point, BMI 18.5–20 kg/m^2^ equals 1 point, and BMI < 18.5 kg/m^2^ generates 2 points. Step 2: calculating and scoring percentage unintentional weight loss. Less than 5% weight loss equals 0 points, weight loss 5–10% equals 1 point, and ≥ 10% generates 2 points. Step 3: score degree of acute disease effect and nutritional intake. Acute illness and no nutritional intake for > 5 days generate 2 points. Step 4: adding points from steps 1–3 to decide overall risk of malnutrition. A total of 0 points = low risk, 1 point = moderate risk, and 2 points or more = high risk. Step 5 calls on action using local guidelines to develop a care plan.

### Body composition

To estimate FFM, we used the medical body composition analyzer Seca 525 (SECA mBCA525) with the software Seca analytics 115 (Seca GmbH & Co., Hamburg, Germany). SECA mBCA525 is a segmental multifrequency BIA-apparatus using the 8-electrode method to measure body composition (17). The multifrequency Seca mBCA 525 used in our study has not been validated among individuals with SCI. However, it shares the same properties with the BIA method employed in the study by Panisset et al. (19). Accurate measures of FFM are derived from TBW, assuming a hydration fraction for FFM of 0.732 (23). To predict impedance and fat mass (FM) by BIA, data on weight, height, and waist circumference are required. Measurement of waist circumference was done by the dietitian (HBS) and carried out with the patient in a supine position, using a standardized measuring tape mid-abdominal between the lower costae and iliac crest. Measurements were performed in a fasted state; patients were instructed to not consume food/drinks or exercise for at least 8 h prior to the measurement, empty their bladder in the morning, and bedrest at least 10 min before the measurement. During the measurement, arms were separated about 30° from the trunk, and legs were separated by about 45°. We used the manufacturers’ estimation of FFM and compared this with FFM calculated with the SCI-specific BIA equation suggested by Kocina et al. (20, 24). To calculate the FFM, we used raw BIA data from the SECA mBCA 525 device and the SCI FFM-equation:

18.874 + Ht^2^/R (0.367) + Wt (0.253) – age (0.081) – sex (5.384) (male = 0, female = 1)

in which Ht^2^ = height in cm squared, R = resistance, and Wt = weight in kg. Fat-free mass index (FFMI) was obtained by the equation:

FFM (kg)/height (m^2^).

FM and FM percentage (FM%) were obtained by the equation:

Body weight (kg) – FFM SECA (kg)/(kg/100).

Suggested SCI-specific BMI obesity cutoff at 22 kg/m^2^ and FM percentage from the BIA measurement were used to describe the prevalence of overweight/obesity in our study population (23).

### Phenotypic and etiologic criteria

All patients were assessed with the GLIM-malnutrition criteria presented in the GLIM consensus report, regardless of risk screening result (14). To achieve a malnutrition diagnosis, at least one phenotypic criterion and one etiologic criterion are required. The following phenotypic criteria were used: non-volitional weight loss of > 5% within past 6 months or > 10% beyond 6 months, low BMI (≤ 20 kg/m^2^ if < 70 years or ≤ 22 kg/m^2^ if ≥ 70 years). FFMI ≤ 17 kg/m^2^ for men and ≤ 15 kg/m^2^ for women were interpreted as low muscle mass, as suggested in the European Society for Clinical Nutrition and Metabolism consensus statement (25). The SCI-specific BIA equations (Kocina) were used to determine FFMI. To assess the first etiologic criteria, reduced food intake, or assimilation, any reduction in food intake for the last 2 weeks upon admission was used. This information was obtained by nurses through routine nutritional risk assessment upon admission and further confirmed during data collection by dietitian interviews. Patients were asked if they had experienced reduced food intake in the 10–14 days leading up to admission for rehabilitation. If it was unclear whether they met their nutritional needs, this was checked through a 3-day food diary. Patients on full enteral feeding meeting their estimated energy needs preceding the time of assessment were interpreted as normally fed. To assess the second etiologic criterion, inflammation, and disease burden, elevated C-reactive protein (CRP) ≥ 5 mg/dL was counted as inflammation. CRP was routinely collected by blood sampling during hospitalization.

### Malnutrition diagnosis

Severity grading of malnutrition was determined based on the phenotypic criteria. To obtain a moderate malnutrition diagnosis, the following phenotypic criterion is required: 5–10% weight loss within past 6 months or > 10–20% beyond 6 months or ≤ 20 kg/m^2^ if < 70 years or ≤ 22 kg/m^2^ if ≥ 70 years, or mild to moderate muscle mass deficit (women ≤ 15 kg/m^2^ FFMI and men ≤ 17 kg/m^2^ FFMI). To obtain a severe malnutrition diagnosis, the following phenotypic criterion is required: > 10% within past 6 months or > 20% beyond 6 months or ≤ 18.5 BMI if < 70 year/< 20 if ≥ 70 years, or severe muscle mass deficit (women ≤ 15 kg/m^2^ FFMI and men ≤ 17 kg/m^2^ FFMI). The BIA Kocina equation was applied across the whole sample to establish the FFMI cutoff. Lower BMI cutoff according to GLIM is ≤ 18.5 kg/m^2^ or ≤ 20 kg/m^2^ if ≥ 70 years.

### Statistics

Data are presented as mean and standard deviation, median and 25–75 percentiles, or frequencies and percentages, depending on the nature of the data. Categorical data were presented using frequencies. For comparison of categorical data, Pearson Chi-square Test or Fisher’s Exact Test was used. Sensitivity, specificity, and Cohen’s kappa were used to evaluate the agreement between the MUST screening and the GLIM diagnosis using crosstabs. The Cohen’s kappa was interpreted as follows: 0–20 no agreement, 0.21–0.39 minimal, 0.40–0.59 weak, 0.60–0.79 moderate, 0.80–0.89 strong, and ≥ 0.90 as almost perfect agreement (26). Relative agreement of the two estimates of FFMI was determined by intraclass correlation coefficients (ICCs) (two-way random, absolute agreement). A Bland-Altman plot was performed to explore bias (estimated by mean differences), LOA (mean difference ± 1.96 SD), and the presence of outliers in the data. Proportional bias was assessed by analyzing the Pearson’s correlation coefficient and the vertical spread of scatter points for the mean of the two BIA equations (27). One sample *t*-test was used to evaluate mean difference between the two FFMI estimations. All statistical analyses were performed using IBM SPSS Statistics for Windows, version 28 (IBM Corp., Armonk, NY, USA).

## Ethics

The implementation of this study was in accordance with the “Declaration of Helsinki”, and all patients signed the consent form to participate. This study was approved by the Regional Committee of Medical and Health Research Ethics (REK 2017/2443). All patients at risk of malnutrition were further assessed in accordance with the hospital’s procedures.

## Results

### Patient characteristics

Ninety-four patients were eligible to participate in the study, but 16 patients were excluded due to psychological factors, lingual barriers, and severe comorbidity. Twelve patients declined participation. In total, 66 patients were included in the study, 16 women and 50 men. Median time since injury was 37.5 days, ranging from 10 to 450 days. Table 1 describes characteristics of the study population, including age, time since injury, level and severity of the SCI, weight on acute admission, unintentional weight loss, BMI, waist circumference, and FFMI (calculated with the SCI-specific Kocina equation and the manufacturers’ estimation). Mean unintentional weight loss was 5.9% (5.2) at 1-month post-SCI. Mean BMI of all patients was 24.7 (4.2) kg/m^2^. Two patients were classified as underweight (BMI ≤ 18.5 kg/m^2^), and one patient was underweight according to the GLIM age-modified cutoff at ≤ 20 kg/m^2^, ≥ 70 years. A total of 18 patients were classified as overweight (BMI ≥ 25.0–29.9 kg/m^2^), and five were obese according to BMI ≥ 30.0–34.9 kg/m^2^. According to the suggested SCI-specific obesity BMI cutoff at 22 kg/m^2^, 49 patients (74%) were overweight. Forty-six patients (39 men and 7 women) had a FM percentage (measured by BIA) corresponding to obesity (SCI cutoff ≥ 22% FM men and ≥ 35% FM women) (28).

**Table 1:** Patient characteristics and injury characteristics for the whole study population (*N* = 66) and the GLIM positive patients (*N* = 41).

Characteristic	Study population	GLIM positive
	Mean (± SD)	Mean (± SD)
Age at injury (years)	51.4 (17.4)	49.9 (17.9)
Gender, *n* (male/female)	50/16	32/9
Time since injury (months)^a^	1.3 (0.3,15)	1.3 (0.5,15)
Time since injury (days)^a^	37.5 (10,450)	38 (15,450)
Weight on acute admission (kg)^b^	78.4 (14.6)	75.4 (13.0)
Weight loss kg/%^c^	5.7 (4.4) / 6.8 (5.0)†	7.3 (4.5) / 8.8 (4.8)
BMI (kg/m^2^)^d^	24.7 (4.2)	23.5 (3.1)
**BMI categories**	**N**	**N**
< 18.5 kg/m^2^	2	2
18.5-25 kg/m^2^	41	29
25-30 kg/m^2^	18	9
> 30 kg/m^2^	5	1
Waist circumference, supine (cm) males/females^e^	95.4 (12.3) / 88.0 (14.8)	92.6 (11.6) / 78.4 (6.7)
FFMI kg/m^2^ males/females. manufacture^f^	18.1 (2.3) / 15.2 (1.1)	17.2 (2.0) / 14.8 (1.0)
FFMI kg/m^2^ males/females. Kocina^g^	17.3 (1.9) / 15.3 (1.6)	17.3 (1.7) / 14.4 (1.0)
Fat mass % males/females, manufacturers^h^	25.8 (8.1) / 34.6 (10.0)	26.3 (7.8) / 30.9 (7.0)
Fat mass % males/females, Kocina^i^	26.6 (6.3) / 35.2 (6.5)	25.7 (5.9) / 33.0 (6.1)
**Etiology and severity of SCI**	**N**	**N**
Traumatic	57	35
Nontraumatic	9	5
**Neurological assessment^α^**		
C1-4AIS A, B and C	9	5
C5-8 AIS A, B and C	9	6
T1-S3 AIS A, B and C	17	12
AIS D at any injury level	31	17

a Time since injury; months/days between time of injury and baseline data, median (range) non-normal distribution.

b Weight on acute admission.

c Weight loss; difference between weight at baseline and weight at time of injury (kg/%).

d Body mass index (BMI) (kg/m^2^) on acute admission.

e Waist circumference measured in a supine position, *n* = 2 missing data.

f Fat-free mass index (FFMI) estimated by bioimpedance analysis (BIA) with the manufacturers equation (SECA), *n* = 2 missing data.

g Fat-free mass index (FFMI) calculated with a spinal cord injury (SCI)-specific equation (Kocina) based on raw data from the BIA measurment. *n* = 2 missing data.

h Fat mass % estimated by bioimpedance analysis (BIA) with the manufacturers equation (SECA). *n* = 2 missing data.

i Fat mass % calculated with a spinal cord injury (SCI)-specific equation (Kocina) based on raw data from the BIA measurment. *n* = 2 missing data.

† , Nine patients had a weight gain.

α , American Spinal Injury Association (ASIA) impairment scale (AIS).

### Malnutrition diagnosis according to GLIM

**Fig. 1:**
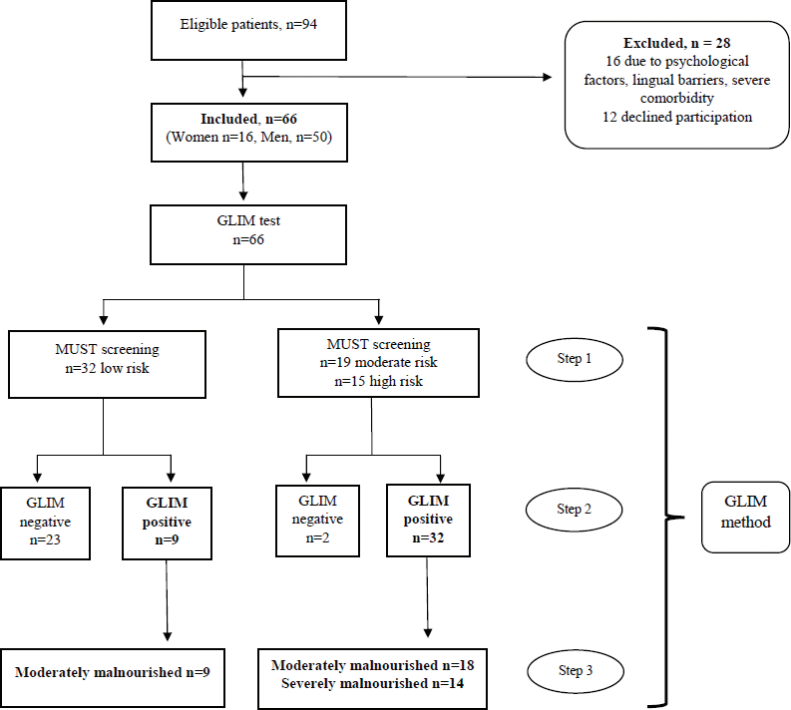
Flow diagram of the study. Patient recruitment was performed from 2018 to 2023. Out of 94 eligible patients, 28 were excluded due to psychological factors, lingual barriers, severe comorbidity, or refusal to participate in this study. Finally, 66 patients were included in this study. Step 1: MUST screening was performed by nurses at the ward and then quality checked by the researcher/dietitian. Step 2: interpreting phenotypic and etiologic criteria. Step 3: determining the severity level of the malnutrition diagnosis, which was performed by the researcher/dietitian. A total of 27 patients were diagnosed as moderately malnourished, and 14 patients had a severe malnutrition diagnosis. GLIM, Global Leadership Initiative on Malnutrition Criteria; MUST, Malnutrition Screening Tool.

In total, 41 patients (62%) were diagnosed as malnourished according to the GLIM criteria, 27 patients were categorized with moderate malnutrition and 14 patients had severe malnutrition (Table 2). The MUST screening revealed 19 patients at moderate risk and 15 at high risk of malnutrition, while 32 patients were assessed as having low risk (Fig. 1). Nine of the 32 patients with low risk according to the MUST were classified as moderately malnourished according to GLIM (Fig. 1). The only phenotypic criteria of the nine patients were low FFMI (Table 3). Four of these nine patients had a combination with the etiological criteria low food intake and inflammation (Table 3). One of the four patients were assessed 450 days post-injury as part of follow-up in the initial pilot study and had a combination of low FFMI and inflammation. Two patients were screened to “moderate risk” according to the MUST but had no malnutrition diagnosis according to GLIM. One of the two patients had a moderate weight loss and low FFMI, but no etiologic criteria, resulting in no GLIM diagnosis. The other individual had no weight loss, but suboptimal FFMI according to the manufacturers’ BIA equation, in addition to low food intake and inflammation. The same individual had a normal FFMI using the SCI-specific BIA equation, resulting in no phenotypic criteria and therefore no GLIM diagnosis. Table 1 describes characteristics of the 41 individuals with a GLIM diagnosis.

**Table 2:** GLIM^a^ screening, assessment, diagnosis, and grading of malnutrition in our population (*N* = 66).

GLIM^a^ steps	*N*
**Step 1: Screening**	
0 = low risk	32
1 = medium risk	19
> 2 high risk	15
**Step 2: Assessment criteria**	
*Phenotypic criteria, any*	**44**
Weight loss^b,c^	33
Low body mass index (kg/m^2^)^d^	3
Reduced muscle mass^e^	26†
*Etiologic criteria, any*	**64**
Reduced food intake or assimilatoc^f^	30
Inflammation^g^	56
**Step 3: Severity**	
Moderate malnutrition^h^	27
Severe malnutrition^i^	14

a Global Leadership Initiative on Malnutrition.

b Moderate weight loss: 5–10% within past 6 months or > 10–20% beyond 6 months.

c Severe weight loss: > 10% within 6 months or > 20% beyond 6 months.

d Body mass index (BMI) < 18.5 kg/m2 if < 70 years, or < 20 kg/m2 if > 70 years.

e Fat-free mass index (FFMI) < 17.0 kg/m2 for men and < 15.0 kg/m2 for women.

f any reduction in food intake for > 2 weeks.

g acute disease/injury or chronic disease-related. Severe inflammation associated with trauma, major infection, burns or closed head injury. High C-reactiv protein (CRP) if combined with any of the inflammation states mentioned.

h Requires 1 phenotypic criterion; 5–10% within past 6 months or > 10–20% beyond 6 months or < 20 BMI if < 70 yr/< 22 if > 70 yr, or mild to moderate muscle mass deficit.

i Requires 1 phenotypic criterion; > 10% within past 6 months or > 20% beyond 6 months or < 18.5 BMI if < 70 yr/< 20 if > 70 yr, or severe muscle mass deficit.

† , *n* = 2 missing.

**Table 3:** Individual combinations of phenotypic and etiologic criteria according to GLIM^a^ (*n* = 66).

ID	Phenotypic				Etiologic		GLIM diagnosis	MUST^j^
	Low BMI^b^	Weight loss 5–10%^c^	Weight loss > 10%^d^	Low FFMI^e^	Low Food intake^f^	Inflammation^g^	Severity level^h,i^	Risk score
1			**X**			**X**	*severe*	*high*
2			**X**	**X**	**X**		*severe*	*high*
3			**X**			**X**	*severe*	*high*
4		**X**		**X**		**X**	*moderate*	*moderate*
5				**X**	**X**	**X**	***moderate***	***low***
6		**X**		**X**		**X**	*moderate*	*high*
7		**X**		**X**	**X**	**X**	*moderate*	*moderate*
8				**X**		**X**	***moderate***	***low***
9		**X**		**X**		**X**	*moderate*	*moderate*
10				**X**	**X**	**X**	***moderate***	***low***
11			**X**		**X**	**X**	*severe*	*high*
12			**X**	**X**	**X**	**X**	*severe*	*high*
13	**X**		**X**	**X**	**X**	**X**	*severe*	*high*
14				**X**		**X**	***moderate***	***low***
15			**X**		**X**		*severe*	*high*
16		**X**		**X**		**X**	*moderate*	*moderate*
17		**X**				**X**	*moderate*	*moderate*
18		**X**				**X**	*moderate*	*moderate*
19		**X**		**X**	**X**	**X**	*moderate*	*moderate*
20			**X**		**X**	**X**	*severe*	*severe*
21		**X**			**X**	**X**	*moderate*	*moderate*
22	**X**	**X**		**X**		**X**	*moderate*	*moderate*
23			**X**	**X**	**X**	**X**	*severe*	*severe*
24	**X**			**X**	**X**	**X**	*moderate*	*moderate*
25				**X**	**X**		***moderate***	***low***
26			**X**		**X**	**X**	*severe*	*high*
27		**X**				**X**	*moderate*	*moderate*
28		**X**		**X**	**X**	**X**	*moderate*	*moderate*
29		**X**			**X**	**X**	*moderate*	*moderate*
30			**X**		**X**	**X**	*severe*	*high*
31				**X**		**X**	***moderate***	***low***
32				**X**		**X**	***moderate***	***low***
33				**X**	**X**	**X**	***moderate***	***low***
34			**X**	**X**		**X**	*severe*	*high*
35			**X**		**X**		*severe*	*high*
36		**X**			**X**	**X**	*moderate*	*moderate*
37		**X**				**X**	*moderate*	*moderate*
38		**X**				**X**	*moderate*	*moderate*
39				**X**	**X**	**X**	***moderate***	***low***
40			**X**	**X**		**X**	*severe*	*high*
41				**X**		**X**	***moderate***	***low***

Bold = individuals with low malnutrition risk and positive GLIM diagnosis.

a Global Leadership Initiative on Malnutrition.

b Body mass index (BMI) < 18.5 kg/m^2^ if < 70 years, or < 20 kg/m^2^ if > 70 years.

c Moderate weight loss: 5–10% within past 6 months or > 10–20% beyond 6 months.

d Severe weight loss: > 10% within 6 months or > 20% beyond 6 months.

e Fat-free mass index (FFMI) < 17.0 kg/m^2^ for men and < 15.0 kg/m^2^ for women.

f any reduction in food intake for > 2 weeks.

g acute disease’injury or chronic disease-related. Severe inflammation associated with trauma,

m ajor infection, bums or closed head injury. High C-reactiv protein (GRP) if combined with any of the inflammation states mentioned.

h Requires 1 phenotypic criterion: 5–10% within past 6 months or > 10–20% beyond 6 months or < 20 BMI if < 70 yr/< 22 if > 70 yr, or mild to moderate muscle mass deficit.

i Requires 1 phenotypic criterion: > 10% within past 6 months or > 20% beyond 6 months or < 18.5 BMI if < 70 yr/< 20 if > 70 yr, or severe muscle mass deficit.

j Malnutrition Universal Screening Tool.

The screening tool MUST showed moderate agreement with GLIM diagnosis (Cohen’s kappa 0.66), with a sensitivity of 0.78 and a specificity of 0.92 compared to GLIM diagnosis.

The crucial variable resulting in GLIM-malnutrition diagnosis was moderate or severe weight loss. Table 3 illustrates the individual combinations of phenotypic and etiologic criteria with GLIM diagnosis and malnutrition risk in all 66 patients.

### FFM estimations

**Fig. 2:**
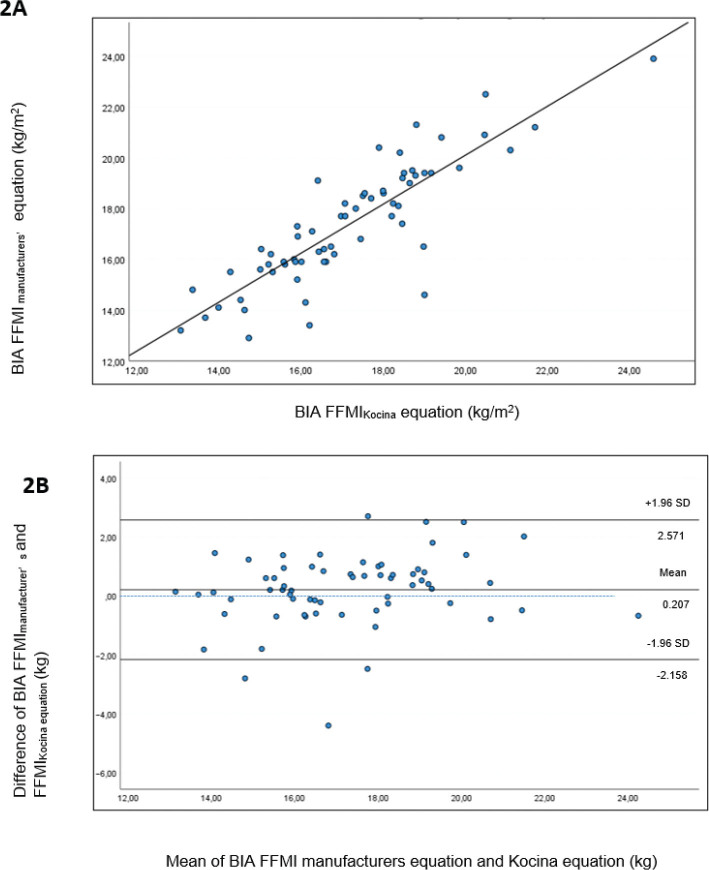
Fat-free mass index (FFMI) assessed by whole-body BIA. (A) Scatter plot for manufacturer’s and Kocina equation FFMI (kg/m^2^) estimates (*R*^2^ = 0.047, SEE = 1.19). Pearson’s correlation coefficient *r* (*P*-value), 0.86 (*P* < 0.001). The solid line represents the regression line between mean FFMI_manufacturer’s_ and FFMI_Kocina_. (B) Bland-Altman plot to show absolute agreement between FFMI estimates of FFMI (kg/m^2^) from the manufacturer’s and Kocina equation. The solid line represents the mean difference of 0.207 kg, with dotted lines representing 95% LOA (± 1.96 SD) of −2.158 to 2.581 kg (*P* = 0.086).

The manufacturer’s BIA equation and the SCI-specific Kocina equation were used to calculate FFMI. There was a strong linear correlation *r* = 0.86 (*P* < 0.001) between the two equations (Fig. 2A), and the relative agreement was excellent (ICC = 0.92, two-way random). The manufacturer’s equation tended to overestimate the FFMI compared to the Kocina equation (mean difference 0.207 kg, 95% CI, −0.09 – 0.501, *P* = 0.087). The Bland-Altman plot (Fig. 2B) illustrates no proportional bias (*P* = 0.086) and moderate LOA ± 2.36 kg.

## Discussion

### Malnutrition diagnosis after SCI

In this cross-sectional study, almost two-thirds of our SCI patients was diagnosed as either moderately or severely malnourished according to the GLIM criteria. We found a moderate agreement between the screening tool MUST and the GLIM diagnosis. However, nine patients (14%) were “false” negative, that is, according to the MUST screening tool, they were categorized as having low risk of malnutrition, while according to the GLIM criteria, they had a moderate malnutrition diagnosis. All the nine patients were diagnosed with malnutrition due to the phenotypic criteria of low FFMI in combination with the etiologic criteria of reduced food intake and/or inflammation. CRP was used as a simple measure of inflammation status. CRP is an acute-phase reactant, which increases following acute traumatic SCI due to a cascade of inflammatory reactions (29). Inflammation is one of the main drivers of disease-related malnutrition and is therefore relevant in the acute/subacute assessment of malnutrition following SCI (30). MUST screening was part of the routine clinical assessment at our hospital ward and was therefore used in this study. We cannot rule out that a more SCI-specific screening tool could have led to different results. The spinal nutrition screening tool (SNST) is a more specific malnutrition screening tool developed by SCI dietitians in the UK, and includes weight loss, BMI adjusted to SCI, age, neurological level of injury, other medical conditions, diet, appetite, and ability to self-feed. In a study by Wong et al. on 150 SCI patients, the SNST showed good validity compared to MUST (sensitivity 86% and specificity 76%) (12). This indicates that MUST is a valid tool to assess malnutrition risk in the SCI population. In a study by Flury et al., they used the SNST at discharge and 3 months post-SCI to calculate malnutrition risk (31). Out of 252 SCI patients, 62% had a malnutrition risk at 3 months post-injury and 40% at discharge (31). Our results support previous reports on malnutrition risk among SCI individuals, and this study highlights that the malnutrition risk can be elevated even above the acute phase.

Current malnutrition diagnostics tools do not consider the obligatory weight and muscle loss after SCI. One could argue that the SCI itself puts the body into a malnourished state with involuntary weight loss and muscle atrophy. Thus, the validity of using the GLIM criteria as gold standard diagnostics criteria in the SCI population can be questioned. Ideally, monitoring tissue loss above the lesion level, including individual energy needs and diet, could give a more precise estimation of malnutrition risk and diagnosis after SCI. Moreover, this would enable more targeted nutrition interventions. However, techniques for accurately measuring the distribution of body tissue, such as magnetic resonance imaging analysis and computed tomography, are costly and have limited availability in the clinical setting.

Using the whole GLIM process provides valuable information on nutritional status at the individual level and adds value in terms of choosing actions to improve nutritional status. As earlier mentioned, nine patients were classified with “low risk” in step 1 of the GLIM process (screening) and would be undiagnosed in an ordinary clinical setting. As we performed all steps of GLIM on all our included patients, we found all nine patients to be moderately malnourished due to low FFMI and a combination of low food intake and/or inflammation, but no weight loss. The low FFMI could be due to the irreversible consequences of SCI, which includes skeletal muscle atrophy and loss of FFM (4, 32). This aspect likely pertains in the chronic phase of an SCI and is particularly plausible for the individual who was evaluated 450 days post-SCI, presenting with both low FFMI and inflammation. Elevated CRP levels are commonly observed in chronic SCI, attributed to clinical factors such as pressure ulcers, urinary tract infections, lack of physical activity, and accumulation of adipose tissue (33, 34). Therefore, CRP is probably less sensitive as a criterion for malnutrition in the chronic phase of SCI. However, four of these patients also reported a low food intake, and a poor diet could partly explain the low FFMI. Indeed, individuals with SCI are at risk of poor dietary intake throughout all phases of the injury. Regular and individual dietary assessments after SCI are important for targeted interventions and should be included into SCI rehabilitation and follow-up protocols.

### Sarcopenic obesity

All except four patients were normal to obese according to the general BMI scale, despite involuntary weight loss. A suggested obesity cutoff for individuals with chronic SCI is BMI ≤ 22 kg/m^2^, and a large proportion of our study population were obese according to these SCI cutoffs (35). Body FM results measured by BIA confirmed this finding. A study examining FM among athletes with SCI discovered a weak correlation in percentage fat between DXA and the BIA Kocina equation (36). This was attributed to individual differences in hydration levels, and we cannot rule out that this extends to our data, resulting in a potential underestimation of FM from BIA in our study. The combination of low FFM and high FM, termed “obese sarcopenia”, may partly explain the increased cardiometabolic risk among individuals with SCI (28). However, it is important to emphasize that obesity and malnutrition can coexist, and a high pre-injury weight may mask a disease-related malnutrition unless necessary examinations are undertaken. This presents a challenge in diagnosing malnutrition in individuals with SCI and necessitates special measures to address it. This includes guiding and empowering patients to adopt a balanced diet that maintains their initial obligatory weight loss and establishes a new, healthier weight adjusted to the muscle loss. Doing so may help prevent future cardiometabolic health issues.

### Consequences of malnutrition

Consequences of malnutrition in the initial weeks post-SCI are poorly understood (3). However, it is plausible to suggest that malnutrition may negatively impact recovery and muscle mass preservation and increase morbidity. A randomized clinical trial by Kaegi-Braun et al. found significantly higher incidence of adverse clinical outcomes (all-cause mortality, readmissions, respiratory/renal/gastrointestinal failure, cardiovascular events, or decline in functional status) among acutely admitted hospital patients (non-SCI) who were GLIM positive (malnourished) compared to GLIM negative (well-nourished) patients (OR 1.65; 95% CI 1.32–2.06; *P* < 0.001). This association was even stronger for the outcome 30-day mortality. Importantly, they observed a higher reduction of adverse clinical outcomes among GLIM positive patients who received individual nutritional therapy versus standard hospital food (37). Wong et al. found similar results in a prospective study among individuals with SCI newly admitted to UK SCI centers. Here, they used both the SNST and MUST and detected a malnutrition prevalence of 44.6% (12). In a second paper, they found that patients classified as malnourished, or at risk, had a significant association with longer inpatient hospital stay, and greater 12-month mortality (11). Although the duration of inpatient hospital stays and mortality after SCI have multifaceted causes, this study highlights the importance of addressing and monitoring nutritional status after SCI.

As discussed in the previous section, muscle fat infiltration, body weight, and total FM tend to increase over time among individuals with SCI (38, 39). The risk of gaining weight and accumulating FM following SCI is important to consider early in the rehabilitation setting. Importantly, more attention on nutritional support during the malnourished state of SCI and transition to the more “well-nourished” state is required. Methods to accurately assess and treat malnutrition following SCI remain to be explored, including the short- and long-term clinical implications.

### Body composition

A reliable BIA measurement depends on the precise estimation of TBW. It is well known that individuals with SCI have increased extracellular water, which, in turn, affects TBW estimations (18). Panisset et al. used isotope tracer dilution (the reference method for measuring TBW) in a population of subacute SCI to validate estimates of FFM from BIA-based predictive equations developed for the SCI population (19). They found that the Kocina equation showed the best fit; however, it increasingly underestimated FFM as the value of FFM increased, compared to the gold standard isotope dilution technique. In the present study, agreement between the manufacturers FFMI estimates and the Kocina FFMI estimates was good at a group level. It is unclear which equation reflects the true value, as we lack comparison to reference methods of FFM, such as isotope dilution technique, magnetic resonance imaging, or DXA.

## Strengths and limitations

Strengths of this study include a fair number of patients, considering the low annual incidence rate of SCI in Norway. Furthermore, the same dietitian performed all BIA measurements and waist circumference, except for the first 18 patients, which were performed by a master student in clinical nutrition under the supervision of the dietitian. Some limitations need to be addressed. First, height was self-reported and could be a possible bias in the BMI assessment. Weight was obtained in a clinical setting, from different nurses, using two types of scales (wheelchair weight and a portable standing scale). This may have led to bias, which can have influenced the accuracy of the BIA measurements. However, the nurses were instructed on how to weigh the patients, and this was performed under the supervision of the dietitian. Another weakness is that imbalance in extracellular and intracellular fluids may have affected the BIA measurement, leading to estimation bias of FFM in this population. We did not use data on waist circumference and SCI-specific BMI cutoffs for the interpretation of our findings. We carefully considered this issue and concluded not to use SCI-specific BMI as these cutoff values are more applicable to chronic SCI patients. Another consideration was that we intended to apply the GLIM criteria to this population and, therefore, choose the GLIM-recommended cutoff points. MUST is not a screening tool specific to SCI, but it was utilized because it was part of the standard routine assessment in the rehabilitation hospital. Similarly, BIA was selected because it was the only clinically available instrument at our rehabilitation facility and offered an easy, low-cost, bedside method to measure FFM.

A small number of the patients had a longer time since injury, as the first 18 patients were part of a pilot which included a mix of traumatic, non-traumatic, and follow-up SCI individuals, with various time since injury. This prompt questions regarding some of the individual results, as certain malnutrition criteria, such as standard FFMI cutoffs and CRP, are not well-suited for individuals with SCI in the chronic phase. Furthermore, we did not analyze potential gender differences from the BIA assessment due to the absence of reference methods. Finally, the lower FFMI cutoffs established in the GLIM criteria may not be universally applicable to all individuals with SCI, given variations across type of injury, gender, and age.

## Conclusion

In this cross-sectional study, 62% of individuals with subacute SCI were malnourished according to the GLIM criteria. The screening tool MUST showed moderate agreement with the GLIM criteria and did not detect risk of all patients with a malnutrition diagnosis. The clinical implications of these findings need further investigation, and there is a need for an international consensus on criteria for diagnosing malnutrition following SCI. Moreover, our findings suggest that both the manufacturer’s equation and the Kocina equation can be used in a clinical setting to estimate FFM. However, further validation and reliability studies are needed to confirm the accuracy of FFM estimates from BIA among individuals with SCI.

## Data Availability

All additional data are available from the corresponding author upon reasonable request.
